# Cellular dynamics across aged human brains uncover a multicellular cascade leading to Alzheimer’s disease

**DOI:** 10.1002/alz.087108

**Published:** 2025-01-03

**Authors:** Philip L. De Jager

**Affiliations:** ^1^ Columbia University Irving Medical Center and the Taub Institute for Research on Alzheimer’s Disease and the Aging Brain, New York, NY USA

## Abstract

**Background:**

Recent studies have revealed diverse Alzheimer’s disease (AD)‐associated cell states, yet when and how they impact the causal chain leading to AD remains unknown.

**Method:**

To reconstruct the dynamics of the brain’s cellular environment along the disease cascade and to distinguish between AD and aging effects, we built a comprehensive cell atlas of the aged prefrontal cortex from 1.64 million single‐nucleus RNA‐seq profiles. We associated glial, vascular and neuronal subpopulations with AD‐related traits for 437 aged individuals and aligned them along the disease cascade using causal modeling.

**Result:**

We identified two distinct lipid‐associated microglial subpopulations, one associated with amyloid‐β proteinopathy while the other mediated the effect of amyloid‐β in accelerating tau proteinopathy, as well as an astrocyte subpopulation that mediated the effect of tau on cognitive decline. To model the coordinated dynamics of the entire cellular environment, we devised the BEYOND methodology, which uncovered two distinct trajectories of brain aging defined by differing sequences of changes in cellular communities. Our set of prospectively autopsied older individuals is separated into one of two possible trajectories, each associated with progressive changes in specific cellular communities that end with either (1) AD dementia or (2) alternative brain aging.

**Conclusion:**

We provide a cellular foundation for a new perspective on AD pathophysiology that could inform the development and personalization of new therapeutic interventions targeting cellular communities while personalizing clinical management for those individuals on the path to AD or to alternative brain aging.

